# Nerve Blocks in Palliative Care

**DOI:** 10.1038/sj.bjc.6602884

**Published:** 2006-01-10

**Authors:** A P Baranowski

**Affiliations:** 1 National Hospital for Neurology and Neurosurgery, London, UK

It was a pleasure to review this book on nerve blocks in palliative care, written by two renowned specialists, Fiona Hicks, who is a consultant in palliative medicine at Leeds Teaching Hospitals Trust and Karen Simpson, a consultant in pain medicine within the same trust. *Nerve Blocks in Palliative Care* has been composed primarily for those who are not familiar with the full potential and risks of nerve blocks in patients with neoplastic disease. As such, the book is aimed primarily at those involved in the day-to-day specialist care of cancer patients such as palliative care doctors, oncologists and specialist nurses, as opposed to pain medicine anaesthetists. This is not a ‘cook book’ as to how to perform the procedures, rather it is an excellent guide on how and when to refer a patient and as such should probably be compulsory reading for those who assess patients for referral to specialist pain teams.

The book starts with a very general section on the philosophy behind the use of interventional blocks. I was amused by the paragraphs on team issues and communications. Many of the problems that are alluded to in these paragraphs are all too familiar to those of us who perform pain medicine interventions. Reading this book prior to referral would help prevent some of the many misunderstandings that pain specialists come across in our practice of pain medicine. The miracle pain relieving injection with no risks does not exist! Similarly, there is no such thing as a ‘simple injection’. Team members can disagree on what is the best way of managing a particular problem and they need to be involved in discussions, in addition to the patient and the relatives.

There is also a very good chapter on the assessment of pain, which is something that is often poorly performed by the nonpain specialist when you take into account the multiple pain mechanisms that may be present at any one time. There now is a wealth of information about the aetiologies and underlying mechanisms of pain in cancer patients and to try and summarise this in a chapter was obviously difficult. The chapter on pain syndromes covers this area well for the nonpain specialist.

The central part of the book then proceeds to cover individual invasive procedures, ranging from trigger point injections through to the more complex neurolytic blocks and intrathecal infusions. These chapters have involved a significant amount of thought and work. Each technique is introduced by comments on specific indications and contraindications; it could be argued that these are the most important points for a non-specialist in pain medicine to take on board. Understanding the limits of any procedure and the complications that may occur are also vital. The limits and complications need to be discussed by a sympathetic person prior to referral to a pain specialist, as the patient needs to be prepared to participate in a discussion with the pain specialist in greater depth. Currently, it is not uncommon for the pain specialist to have to break the news that severe complications can occur and that there is a failure rate; the myth of the ‘miracle risk free injection’ is broken, which may result in profound disappointment and occasionally increased patient distress.

Finally, the book finishes with a chapter, albeit brief, on the ethics involved in pain medicine and the terminally ill. Once more, we are urged to consider not only the potential benefits but also the risks.

At the end of the day, those performing the procedures are best suited to tackle the issues around interventions for pain management. However, it is important that the potential for improved pain relief and reduced suffering are not missed by those involved in the day-to-day management of cancer patients. Also, patients need to have an understanding of what may be involved prior to meeting the pain team. Recently, I have been asked to give a talk on invasive procedures to our palliative care team. I have responded by asking that they read this book on nerve blocks in palliative care – I feel that would provide them with more education than I could provide in an hour. My talk will be around this excellent book!

